# JNK1 and JNK3: divergent functions in hippocampal metabolic-cognitive function

**DOI:** 10.1186/s10020-022-00471-y

**Published:** 2022-05-04

**Authors:** Oriol Busquets, Triana Espinosa-Jiménez, Miren Ettcheto, Jordi Olloquequi, Mònica Bulló, Eva Carro, José Luis Cantero, Gemma Casadesús, Jaume Folch, Ester Verdaguer, Carme Auladell, Antoni Camins

**Affiliations:** 1 Department of Pharmacology, Toxicology and Therapeutic Chemistry, Pharmacy and Food Sciences Faculty, University of Barcelona, 08028 Barcelona, Spain; 2 Department of Biochemistry and Biotechnology, Medicine and Health Sciences Faculty, University Rovira i Virgili, 43201 Reus, Spain; 3 Centre for Biomedical Research of Neurodegenerative Diseases (CIBERNED), Instituto de Salud Carlos III, 28029 Madrid, Spain; 4 Institut de Neurociències, University of Barcelona, 08035 Barcelona, Spain; 5 Laboratory of Cellular and Molecular Pathology, Facultad de Ciencias de La Salud, Instituto de Ciencias Biomédicas, Universidad Autónoma de Chile, Talca, Chile; 6 Institut d’Investigació Sanitària Pere Virgili (IISPV), Hospital Universitari de Sant Joan de Reus, 43204 Reus, Spain; 7 CIBER de Fisiopatología de la Obesidad y la Nutrición (CIBEROBN), Instituto de Salud Carlos III, 28029 Madrid, Spain; 8 Network Center for Biomedical Research in Neurodegenerative Diseases (CIBERNED), Madrid, Spain; 9 Group of Neurodegenerative Diseases, Hospital Universitario 12 de Octubre Research Institute (imas12), Madrid, Spain; 10 Laboratory of Functional Neuroscience, Pablo de Olavide University, 41013 Seville, Spain; 11 Department of Pharmacology & Therapeutics, College of Medicine, University of Florida, Gainesville, FL 32610 USA; 12 Department of Cell Biology, Physiology and Immunology, Biology Faculty, University of Barcelona, 08028 Barcelona, Spain; 13 Present Address: Dominick P. Purpura Department of Neurosciences, Albert Einstein College of Medicine, New York City, 10461 USA

**Keywords:** JNK1, JNK3, Metabolism, Cognition, High-fat diet

## Abstract

**Background and aim:**

The appearance of alterations in normal metabolic activity has been increasingly considered a risk factor for the development of sporadic and late-onset neurodegenerative diseases. In this report, we induced chronic metabolic stress by feeding of a high-fat diet (HFD) in order to study its consequences in cognition. We also studied the effects of a loss of function of isoforms 1 and 3 of the c-Jun N-terminal Kinases (JNK), stress and cell death response elements.

**Methods:**

Animals were fed either with conventional chow or with HFD, from their weaning until their sacrifice at 9 months. Before sacrifice, body weight, intraperitoneal glucose and insulin tolerance test (IP-GTT and IP‑ITT) were performed to evaluate peripheral biometrics. Additionally, cognitive behavioral tests and analysis of spine density were performed to assess cognitive function. Molecular studies were carried out to confirm the effects of metabolic stressors in the hippocampus relative to cognitive loss.

**Results:**

Our studies demonstrated that HFD in *Jnk3*^*−/−*^ lead to synergetic responses. Loss of function of JNK3 led to increased body weight, especially when exposed to an HFD and they had significantly decreased response to insulin. These mice also showed increased stress in the endoplasmic reticulum and diminished cognitive capacity. However, loss of function of JNK1 promoted normal or heightened energetic metabolism and preserved cognitive function even when chronically metabolically stressed.

**Conclusions:**

Downregulation of JNK3 does not seem to be a suitable target for the modulation of energetic-cognitive dysregulations while loss of function of JNK1 seems to promote a good metabolic-cognitive profile, just like resistance to the negative effects of chronic feeding with HFD.

**Supplementary Information:**

The online version contains supplementary material available at 10.1186/s10020-022-00471-y.

## Introduction

The disruption of physiological energetic metabolism can be the cause for the development of cognitive deficits in the context of neurodegenerative diseases (Muddapu et al. 2020; Procaccini et al. 2016; Cai et al. 2012; Głuchowska et al. 2021; Winkler et al. 2015; Barbagallo 2014; Cereda et al. 2009). Causes for the dysregulations may vary from changes at the cellular level on the functionality of organelles like the mitochondria or endoplasmic reticulum or, severe deviations like the appearance of chronic tissue neuroinflammation (Muddapu et al. 2020; Procaccini et al. 2016) and the loss of sensitivity to hormones like insulin or leptin (Lloret et al. 2019; Forny-Germano et al. 2019; Cai 2013; Doherty 2011; De La Monte 2016; Taouis 2011).

The c-Jun N-terminal Kinases (JNK) are a subfamily of the Mitogen Activated Protein Kinases (MAPK) that have been studied for their role in the regulation of a wide myriad of physiological and disease-related mechanisms, making them appealing targets for the prevention and amelioration of many pathological states (Sabapathy 2012). These kinases are subdivided into three different isoforms (JNK1, JNK2 and JNK3) which have shown redundant and/or divergent functions depending on organ, subcellular localization or metabolic situation (Sabapathy 2012; Coffey et al. 2000; Coffey 2014). From a general standpoint, JNK1 and JNK2 are distributed almost ubiquitously in the body whereas JNK3 is found only in the brain, heart, beta pancreatic cells and testes (Coffey 2014). In the brain, isoform JNK1 shows intermediate to low expression in the cortex, hippocampus and cerebellum, while JNK2 expresses at low levels throughout; JNK3 is the most abundantly expressed isoform in the brain. In the past, our research group and others have presented data on the effects of a modulation of the JNKs in the control of metabolic biomarkers and its consequences in cognition. In these reports, it was demonstrated through different experimental approaches that knockout of isoform JNK2 leads to negative effects in normal metabolism of the hippocampal tissue in mice and it has consequences in cognitive capacity (Busquets et al. 2019a; Raciti et al. 2012). When JNK1 is knocked out, results point to the opposite outcome. In fact, other teams have reported that the downregulation of JNK1 leads to a heightened control of metabolic activity both in the periphery and central tissues, coupled with a resistance of the animals to the negative effects of fat-enriched diets (Sabio et al. 2009, 2010; Becattini et al. 2016; Mohammad et al. 2018). In our own report, we demonstrated for the first time the beneficial effects of this loss of function of JNK1 in multiple biomarkers of cognitive function in the hippocampus of mice (Busquets et al. 2019b). As for JNK3, there are not many published reports on its role in metabolism.

From a general standpoint, JNK3 is an important enzyme in the brain, responsible of the control of brain development, neurite formation and plasticity, as well as regeneration, and differentiation, learning and memory (Yarza et al. 2016). In pathological states, this enzyme has been linked with an overactivation of the JNKs in the brain in situations of ischemia, hypoxia or epilepsy (Yarza et al. 2016) and, its ablation, has been associated with protection against excitotoxicity and apoptotic mechanisms (Yang et al. 1997; Brecht et al. 2005). Problematically, the loss of function of JNK3 seems to have negative consequences. In a report from Vernia et al., they showed that metabolic stress induces hyperphagia in JNK3 KO mice due to its essential role in the control of the response to leptin (Vernia et al. 2016). As pointed by the authors, this data would be evidence to the inadequacy of JNK3 as a target for drug therapy for metabolic-related alterations.

On this report, we initially focused on determining the effects of the loss of function of JNK3 in regulatory pathways studied in the past by our team. Later, we used a TaqMan® Array to uncover changes in the transcriptome caused by the absence of JNK1 and JNK3, as well as the individual and synergistic effects of a chronic feeding with a high-fat diet (HFD). Feeding of a HFD was chosen as a model of metabolic stress and potential contributor to the appearance of sporadic forms of neurodegeneration based on preclinical, clinical and epidemiological reported data (Henneberg and Hoyer 1995; Hoyer et al. 1996; Felice and Lourenco 2015; Grillo et al. 2015; Ott et al. 1999; Willmann et al. 2020; Kaplan et al. 2022; Akinola 2016; Milstein and Ferris 2021; Malan et al. 2021). In the end, the results allowed us to describe further the pathways affected by loss of function of JNK1 and JNK3 and thus, we were able to gather more evidence on their suitability as targets for pharmacological approaches.

## Materials and methods

### Animals

Male 9 months-of-age wild-type C57BL6/J (WT), *Mapk8*^*−/−*^ (JNK1; *Jnk1*^*−/−*^) and *Mapk10*^*−/−*^ (JNK3; *Jnk3*^*−/−*^) mice were used in this study (Dong et al. 1998). They were fed either control (CT) or HFD (45% fat content) ad libitum (Busquets et al. 2019b, c). Diet specifications were as described in Busquets et al. (2017). They were kept in constant conditions of temperature, humidity and 12 h light/dark cycles. Animals were weighed monthly and tested in the insulin tolerance test (ITT; n = 10–12/experimental group) and novel object recognition test (NORT; n = 10–12/experimental group) as previously described (Busquets et al. 2019c). Hippocampal tissue was used for all assays [dissected similarly as described by Sultan (2013)]. Dendritic spines numbers and shapes were analyzed through the Golgi stain in the dentate gyrus of the hippocampus (n = 8/experimental group) (Busquets et al. 2019c). All protocols and procedures followed the bioethics guidelines stablished by the European Communities Council Directive 2010/63/EU.

### TaqMan® array

RNA and cDNA were obtained as previously described from hippocampal samples (n = 4/experimental group) (Busquets et al. 2019c). TaqMan® Array Fast plates (ThermoFisher Scientific, Inc.) were used to analyze a total of 48 genes. These were selected by their described relevance in the regulation of energetic metabolism and cognitive function.

*18S*, *Actb*, *Gapdh*, *Hprt* and *Gusb* were tested as housekeeping genes in all samples. *Hprt* showed the least variability and was thus selected to perform any analysis. Targets: *Slc2a1*, *Slc2a2*, *Slc2a3*, *Slc2a4*, *Insr*, *Irs1*, *Irs2*, *Prkaa1*, *Akt1*, *Akt2*, *Creb1*, *Gsk3β*, *Pparγ*, *Pparγc1α*, *Ptpn1*, *Hk1*, *Hk2*, *Pfkp*, *Pkm*, *Pdha1*, *Pdha2*, *Ndufv1*, *Sdha*, *Sdhb*, *Uqcrc1*, *Uqcrb*, *Cycs*, *Cox4i1*, *Atp5b*, *Sod1*, *Gpx1*, *Cat*, *Bdnf*, *Ntrk2*, *Ppp1r9b*, *Syp*, *Dlg4*, *Nrxn1*, *Nrxn2*, *Nrxn3*, *Nlgn1*, *Nlgn2*, *Nlgn3*. No data was reported of *Slc2a2*, *Pparγ and Pdha2* since the TaqMan® probes produced either no signal or a C_T_ value over 35. Specific descriptions for each of the genes included in the study can be found in Additional file 1: S1.

### Immunoblot

Protein extraction and detection were performed as previously described (n = 4/experimental group) (Busquets et al. 2019c). Fresh brains of at least 4 mice per group were extracted right after euthanasia (cervical dislocation) and hippocampus were dissected and kept frozen at − 80 °C until use. After, samples were homogenized in lysis buffer (Tris HCl 1 M pH 7.4, NaCl 5 M, EDTA 0.5 M pH 8, Triton, distilled H20) containing a protease (Complete Mini, EDTA-free; Protease Inhibitor cocktail tablets, 11836170001, Roche Diagnostics GmbH, Germany) and phosphatase inhibitor cocktail (Phosphatase Inhibitor Cocktail 3, P0044, Sigma-Aldrich, USA). The samples were centrifuged at 14,000 rpm for 10 min at 4 °C after a 30-min incubation at the same temperature. The supernatant was recovered and frozen at − 80 °C until use. Sample protein concentration was determined using the Pierce ™ BCA Protein Assay Kit (Thermo Scientific ™). For immunoblot assays, 10 µg per sample were used and denatured at 95 °C for 5-min in a sample buffer (0.5 M Tris HCl, pH 6.8, 10% glycerol, 2% (w/v) SDS, 5% (v/v) 2-mercaptoethanol, 0.05% bromophenol blue). Electrophoresis was performed on acrylamide gels of 7, 10, and 12% concentration at constant 120 V and transferred to polyvinylidene difluoride sheets (Immobilon®-P Transfer Membrane; IPVH00010; Merk Millipore Ltd., USA) at constant 200 mA for 120 min. Then, membranes were blocked for 1-h with 5% non-fat milk dissolved in TBS-T buffer (0.5 mM Tris; NaCl, Tween® 20 (P1379, Sigma-Aldrich, USA), pH 7.5), washed with TBS-T three times for 5-min and incubated with the appropriate primary antibody, detailed in the text, overnight (O/N) at 4 °C. Subsequently, blots were washed in TBS-T buffer and incubated at room temperature for 1-h with the appropriate secondary antibody. The following antibodies were used: Protein Kinase-like Endoplasmic Reticulum Kinase (PERK; Cell Signaling #3192), P-PERK (Thr980; Cell Signaling #3179), Eukaryotic Initiation Factor 2 α (EIF2; Cell Signaling #9722), P-EIF2 (Ser51; Cell Signaling #9721), Activated Transcription Factor 4 (ATF4; Santa Cruz Biotechnology; sc-200), Inositol-Requiring Enzyme 1 alpha (IRE1α; Santa Cruz Biotechnology; sc-390960), P-IRE1α (Ser724; Nobus Biologicals; NB100-2323), Protein Tyrosine Phosphatase 1 Beta (PTP1B; Millipore ABS40) and P-PTP1B (Ser50; GeneTex; GTX55423). Finally, results were obtained through chemiluminescence detection using the Pierce® ECL Western Blotting Substrate (#32106, Thermo Scientific, USA), a Bio-Rad Universal Hood II Molecular Imager and the Image Lab v5.2.1 software (Bio-Rad laboratories). Measurements were expressed in arbitrary units and all results were normalized with the corresponding loading control (Glyceraldehyde-3-phosphate dehydrogenase; GAPDH).

### Immunofluorescence

Animals (n = 4/experimental group) were anaesthetized through an intraperitoneal injection of ketamine (100 mg/kg) and xylazine (10 mg/kg) before intracardiac perfusion with 4% paraformaldehyde. Posterior brain fixation, sectioning, and labelling have been previously described (Busquets et al. 2019c). When they were in the no-pain sleep phase, they were intracardiacally perfused with 4% paraformaldehyde (PFA) diluted in 0.1 M phosphate buffer (PB). After perfusion, brains were removed and stored in 4% PFA O/N at 4 °C. The next day, the solution was changed into 4% PFA + 30% sucrose. Coronal sections of 20 µm were obtained by a cryostat (Leica Microsystems), kept in a cryoprotectant solution at − 20 °C until their use.

On the 1st day of the assay, free-floating sections were washed three times with 0.1 mol/L PBS pH 7.35 and after, five times with PBS-T (PBS 0.1 M; 0.2% Triton X-100). The, they were blocked in a solution containing 10% fetal bovine serum (FBS) and 1% Triton X-100 diluted with PBS-T five times for 5 min each and incubated with the Glial fibrillary acidic protein (GFAP) antibody (Dako-Z0334). On the 2nd day, slices were washed with PBS-T 5 times for 5 min and incubated with the pertinent secondary antibody for 2 h at room temperature. Finally, sections were treated with 0.1 µg/mL Hoechst (Sigma-Aldrich, St Louis, MO, USA) during 8 min in the dark at room temperature and washed with PBS 0.1 M. All reagents, containers and materials exposed to Hoechst were properly managed and processed to avoid any cytotoxic contamination. Finally, brain slices were mounted in gelatin-coated slides using Fluoromount G (EMS) and were left to dry O/N. Image acquisition was obtained using an epifluorescence microscope (Olympus BX61 Laboratory Microscope, Melville, NY-Olympus America Inc.). Comparative analysis of fluorescence intensity was done as described in Busquets et al. (2017).

### Hippocampal spine density analysis

To carry out the spine density analysis, five animals per group were used which were sacrifice by cervical dislocation. After, brain was isolated and the directions of the Kit purchased from FD Neurotechnologies, Inc. (FD Rapid GolgiStainTM Kit; Cat #PK401) were followed. Images were obtained by BX61 Laboratory Microscope (Melville NY-Olympus America Inc.). The quantification was carried out by selecting five neurons per animal in the dentate gyrus (DG) of the hippocampus. Measurement was done at least 50 μm from the soma along consecutive 10 μm on secondary branches starting 10 μm after branching from the primary dendrite. Spine density was calculated by dividing the number of spines per segment by the length of the segment and was expressed as the number of spines per 10 μm of dendrite.

### Novel Object Recognition Test (NORT)

The Novel Object Recognition Test was used to evaluate hippocampal-dependent recognition memory of mice (Hoyer et al. 1996). The task procedure consisted of three phases: habituation, familiarization and probe. In the habituation phase, mice explored individually a circular open-field arena of 40 cm of diameter for 3 consecutive days, 10 min for each session. On the 4th day (familiarization), each mouse was placed in the arena containing two identical objects (A + A) in the middle of the field for 10 min. To perform the test phase, mice were returned 24 h later to the open-field with two objects, one was identical to the day before and the other was a novel object (A + B) for 10 min. Light intensity was kept constant in all phases and the arena and objects were cleaned with 96° ethanol between each animal to eliminate olfactory cues. Exploration activity was defined as the orientation of snout of the animals toward the object, sniffing or touching. The exploratory analysis was expressed as discrimination index (DI). DI = (novel object exploration time/total exploration time) − (familial object exploration time/total exploration time), measured in seconds.

### Statistical analysis

All statistical analyses and figures were conducted in Graph Pad Prism v9 (Graph Pad Software LLC). Data from the TaqMan® array was presented in a heat map indicating increased expression in red and decreased in green. Other data was presented in bar graphs and/or violin plots showing all data points. All data showed normality and was analyzed using two-way ANOVA and Tukey’s. Significance was indicated as *p < 0.05, **p < 0.01 and ***p < 0.001.

## Results

### *Jnk3*^−/−^ mice fed with HFD show weight increase and insulin resistance together with cognitive decline

**Fig. 1:**
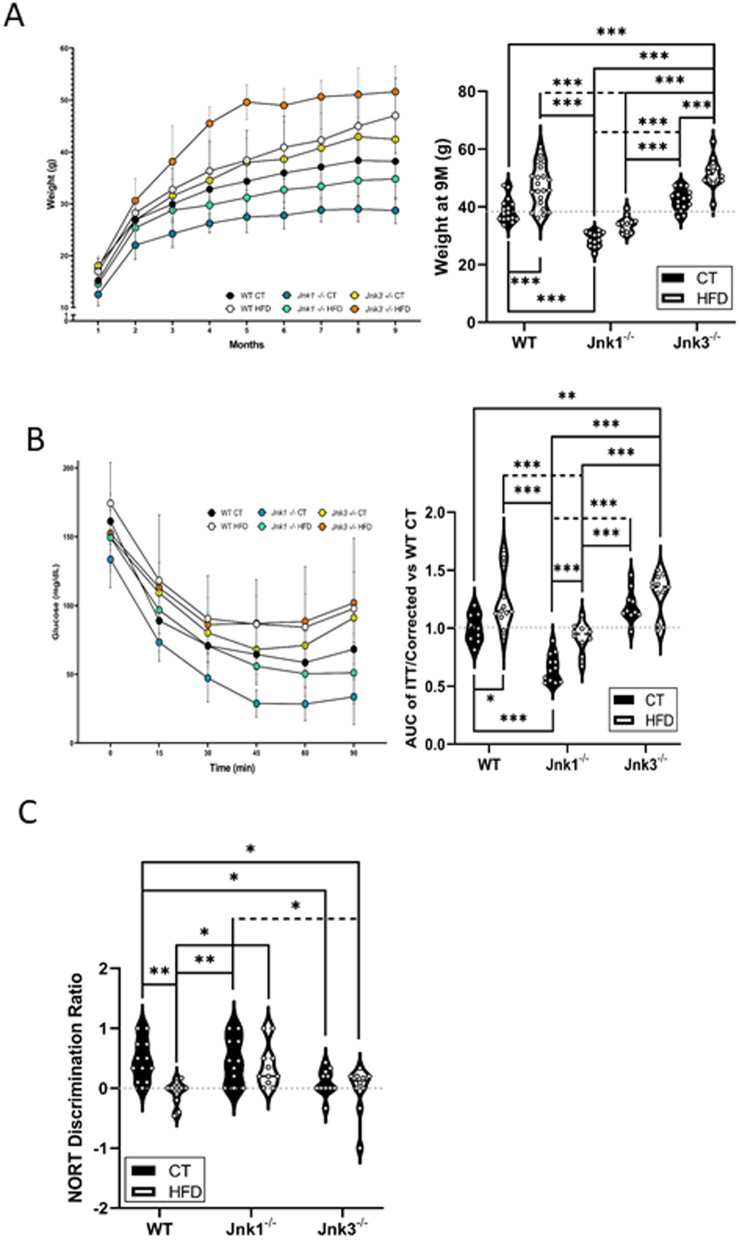
**A** Representation of progression of the weight of the animals included in the study during the 9-month growth period (monthly measurement) (n = 10–21/experimental groups). **B** ITT results were extrapolated into an area under the curve value which was used for the statistical comparison. Results were represented as bar graphs or violin plots. Differences between groups were analyzed using ANOVA and Tukey’s. Significance was represented as follows: *p < 0.05, **p < 0.01 and ***p < 0.001 (n = 10–12/experimental group). **C** Graphical representation of the individual values of the discrimination ratio of the animals in the NORT. Values were calculated by using the following formula: discrimination ratio = (time spent exploring the new object − time spent exploring the known object)/total exploration time. Any animals that showed lower than average activity or any divergent preference for one of the identical objects in the training had their results discarded from the analysis (n = 10–12/experimental group)

Regarding the weight and response to insulin in each experimental group, a significant weight gain in WT and Jnk3^−/−^ animals by 9 months of age was observed after HFD (Fig. 1A; p < 0.001 WT CT vs WT HFD, WT HFD vs Jnk1^−/−^ CT, WT HFD vs Jnk1^−/−^ HFD, WT HFD vs Jnk3^−/−^ HFD, WT CT vs Jnk3^−/−^ HFD, Jnk1^−/−^ CT vs Jnk3^−/−^ HFD, Jnk1^−/−^ HFD vs Jnk3^−/−^ HFD and Jnk3^−/−^ CT vs Jnk3^−/−^ HFD). These animals also showed decreased response to insulin (Fig. 1B; p < 0.05 WT CT vs WT HFD; p < 0.01 WT CT vs Jnk3^−/−^ HFD; WT HFD vs Jnk1^−/−^ CT, WT HFD vs Jnk1^−/−^ HFD, Jnk1^−/−^ CT vs Jnk3^−/−^ HFD, Jnk1^−/−^ HFD vs Jnk3^−/−^ HFD and Jnk3^−/−^ CT vs Jnk3^−/−^ HFD). In contrast, Jnk1^−/−^ animals showed significantly decreased body weight when compared to WT CT (Fig. 1A; p < 0.001 WT CT vs Jnk1^−/−^ CT), just like heightened response to insulin (Fig. 1B; p < 0.001 WT CT vs Jnk1^−/−^ CT). WT and Jnk3^−/−^ HFD animals also showed reductions in their discriminatory capacity when tested through the NORT. Jnk3^−/−^ animals presented decreased discriminatory capacity independently of whether they had been metabolically stressed with HFD or not (Fig. 1C; p < 0.05 WT CT vs Jnk3^−/−^ CT, WT CT vs Jnk3^−/−^ HFD, WT HFD vs Jnk1^−/−^ HFD and Jnk1^−/−^ CT vs Jnk3^−/−^ HFD; p < 0.01 WT CT vs WT HFD and WT HFD vs Jnk1^−/−^ CT).

### *Jnk1*^−/−^ and *Jnk3*^−/−^ mice show divergent genetic expression profiles in genes related to energetic metabolism and maintenance of cognitive function

**Fig. 2:**
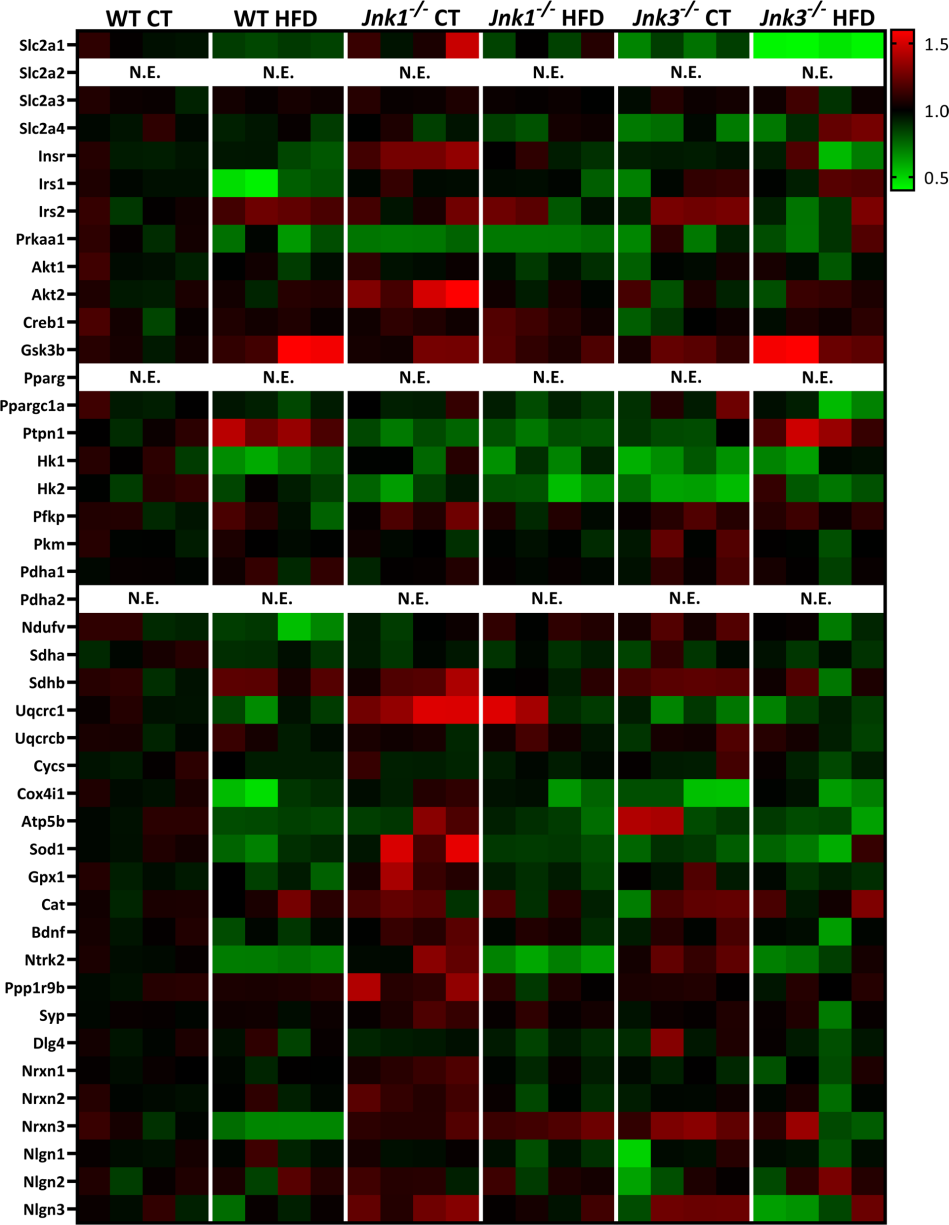
Heat map of the individual results of the TaqMan® array used in this study. Genes were organized under specific subgroups according to which pathway they belong to. Differences in genetic expression were calculated against the control experimental group. No data was reported for *Slc2a2*, *Pparγ and Pdha2* since the TaqMan® probes produced either no signal or a C_T_ value over 35. *N.E.* not expressed (n = 4/experimental group)

Results derived of the array were presented in a Heat Map (Fig. 2) in which the individual values of each of the samples were represented (n = 4/experimental group). Significant results were highlighted and analyzed in posterior figures, together with additional collected data.

**Fig. 3:**
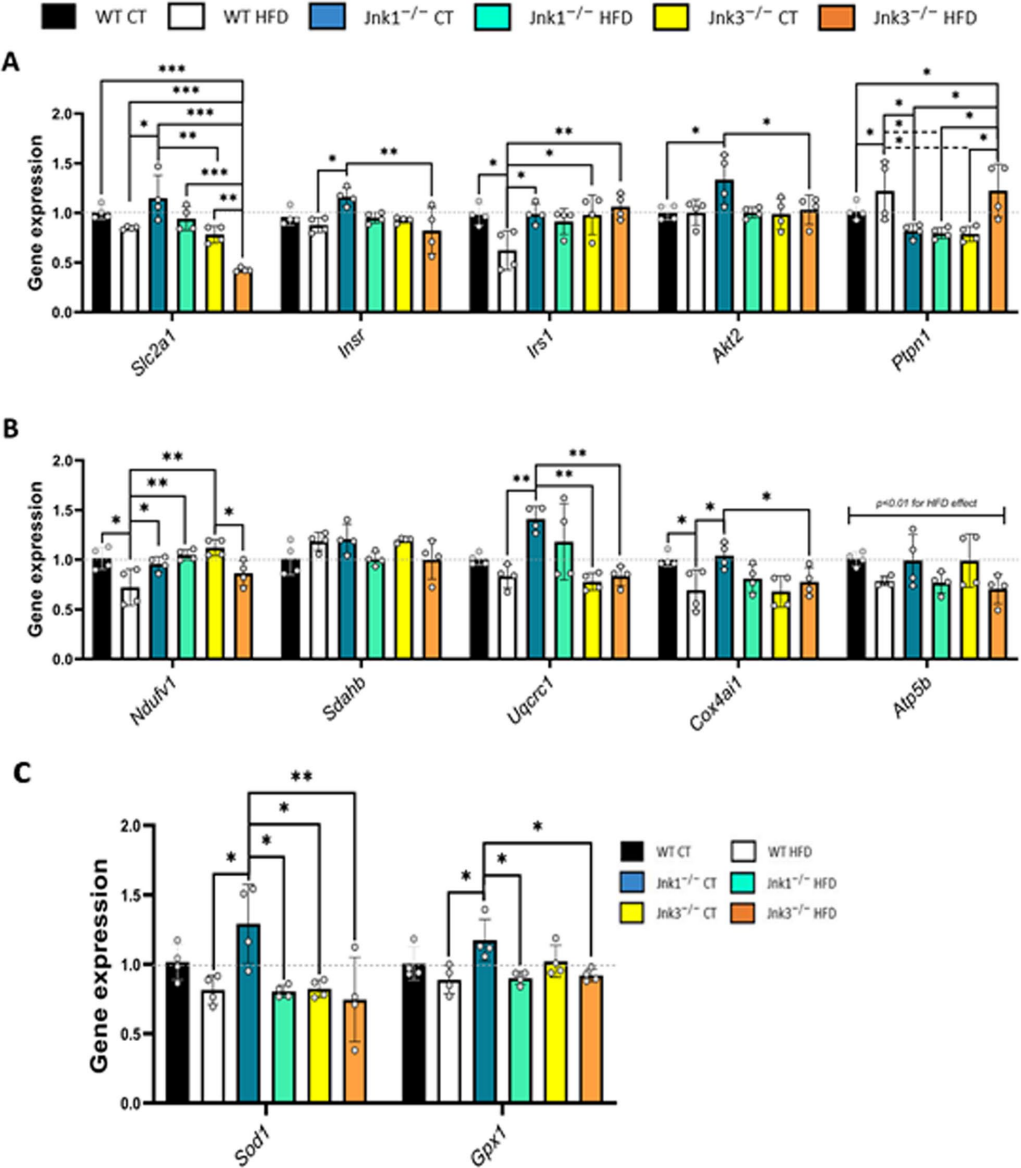
Loss of function of JNK1 and JNK3 cause changes in the transcriptomic profile of genes linked to: **A** glucose uptake and insulin signaling, **B** mitochondrial electron transport chain and **C** antioxidant enzymes (n = 4 per experimental group). Results were represented as bar graphs. Differences between groups were analyzed using ANOVA and Tukey’s. Significance was represented as follows: *p < 0.05, **p < 0.01 and ***p < 0.001

The first subset of targeted genes in the array that showed significant differences in the expression profile were involved in energetic metabolism, including glucose transporters, glycolysis (Fig. 3A), and mitochondrial function (Fig. 3B) and also gene expression of antioxidant enzymes (Fig. 3C). Changes were observed in the glucose transporter 1 (*Slc2a1*), insulin receptor (*Insr*), insulin receptor substrate 1 (*Irs1*), protein kinase Akt 2 (*Akt2*), as well as the protein phosphatase *Ptpn1*. Overall, data indicated that the combination of the loss of function of JNK3 induced mild downregulation of *Slc2a1* which, was aggravated with long-term HFD feeding. Also, HFD feeding promoted significant upregulation of the expression of *Ptpn1* both in WT and *Jnk3*^*−/−*^ HFD conditions. *Jnk1*^*−/−*^ animals showed increased expression on *Slc2a1*, *Insr* and *Akt2* (Fig. 3A; *Slc2a1*: *p* < 0.05 WT HFD vs *Jnk1*^*−/−*^ CT; *p* < 0.01 *Jnk1*^*−/−*^ CT vs *Jnk3*^*−/−*^ CT and *Jnk3*^*−/−*^ CT vs *Jnk3*^*−/−*^ HFD; *p* < 0.001 WT CT vs *Jnk3*^*−/−*^ HFD, WT HFD vs *Jnk3*^*−/−*^ HFD, *Jnk1*^*−/−*^ CT vs *Jnk3*^*−/−*^ HFD and *Jnk1*^*−/−*^ HFD vs *Jnk3*^*−/−*^ HFD; *Insr*: *p* < 0.05 WT HFD vs *Jnk1*^*−/−*^ CT; *p* < 0.01 *Jnk1*^*−/−*^ CT vs *Jnk3*^*−/−*^ HFD; *Irs1*: *p* < 0.05 WT CT vs WT HFD, WT HFD vs *Jnk1*^*−/−*^ CT and WT HFD vs *Jnk3*^*−/−*^ CT; *p* < 0.01 WT HFD vs *Jnk3*^*−/−*^ HFD; *Akt2*: *p* < 0.05 WT HFD vs *Jnk1*^*−/−*^ CT and *Jnk1*^*−/−*^ CT vs *Jnk3*^*−/−*^ HFD; *Ptpn1*: *p* < 0.05 WT CT vs WT HFD, WT CT vs *Jnk3*^*−/−*^ HFD, WT HFD vs *Jnk1*^*−/−*^ CT, WT HFD vs *Jnk1*^*−/−*^ HFD, WT HFD vs *Jnk3*^*−/−*^ CT, *Jnk1*^*−/−*^ CT vs *Jnk3*^*−/−*^ HFD, *Jnk1*^*−/−*^ HFD vs *Jnk3*^*−/−*^ HFD, *Jnk3*^*−/−*^ CT vs *Jnk3*^*−/−*^ HFD). In the mitochondrial-related subset of genes, HFD was observed to have downregulating effects in some of the genes coding for the subunits of the OXPHOS complexes in WT and *Jnk3*^*−/−*^ conditions. The most significant effects caused by HFD were detected on the *Atp5b* subunit of complex V, in which all experimental groups exposed to HFD showed downregulation. *Jnk1*^*−/−*^ CT animals showed control like levels of expression in most cases except on *Uqcrc1* (subunit of complex III) which was significantly upregulated (Fig. 3B; *Ndufv1*: *p* < 0.05 WT CT vs WT HFD, WT HFD vs *Jnk1*^*−/−*^ CT and *Jnk3*^*−/−*^ CT vs *Jnk3*^*−/−*^ HFD; *p* < 0.01 WT HFD vs *Jnk1*^*−/−*^ HFD, WT HFD vs *Jnk3*^*−/−*^ CT; *Uqcrc1*: *p* < 0.01 *Jnk1*^*−/−*^ CT vs WT HFD, *Jnk1*^*−/−*^ CT vs *Jnk3*^*−/−*^ CT and *Jnk1*^*−/−*^ CT vs *Jnk3*^*−/−*^ HFD; *Cox4ai1*: *p* < 0.05 WT CT vs WT HFD, WT HFD vs *Jnk1*^*−/−*^ CT and *Jnk1*^*−/−*^ CT vs *Jnk3*^*−/−*^ HFD; *Atp5b*: *p* < 0.05 for HFD effect). Finally, detection of changes of antioxidant genes enzymes *Sod1* and *Gpx1* indicated an expression increased on *Jnk1*^*−/−*^ CT animals (Fig. 3C; *Sod1*: *p* < 0.05 WT HFD vs *Jnk1*^*−/−*^ CT, *Jnk1*^*−/−*^ CT vs *Jnk1*^*−/−*^ HFD, *Jnk1*^*−/−*^ CT vs *Jnk3*^*−/−*^ CT and *Jnk1*^*−/−*^ CT vs *Jnk3*^*−/−*^ HFD; *Gpx1*: *p* < 0.05 WT HFD vs *Jnk1*^*−/−*^ CT, *Jnk1*^*−/−*^ CT vs *Jnk1*^*−/−*^ HFD and *Jnk1*^*−/−*^ CT vs *Jnk3*^*−/−*^ HFD).

**Fig. 4:**
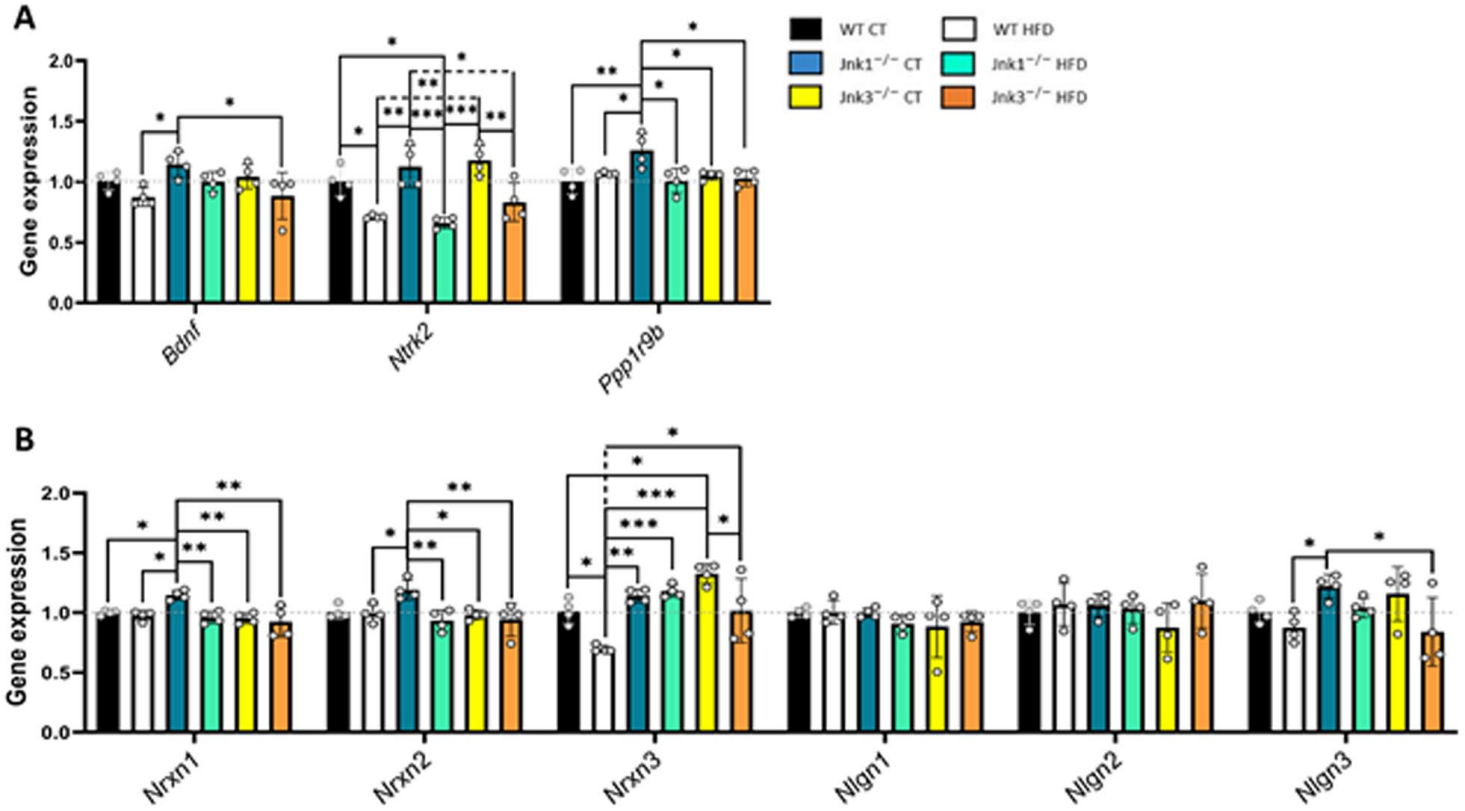
Hippocampal cognition biomarkers are affected by loss of JNK1 and JNK3. **A** and **B** Changes in Cognition-related genes (n = 4 per experimental group). Results were represented as bar graphs. Differences between groups were analyzed using ANOVA and Tukey’s. Significance was represented as follows: *p < 0.05, **p < 0.01 and ***p < 0.001

The second subset of genes included in the array were responsible for the control and maintenance of proper cognitive activity in neuronal and synaptic structures (Fig. 4A, B). It was observed that, HFD promoted a significant downregulation of the neurotrophin receptor tyrosine kinase gene *Ntrk2* in all experimental groups, which was paired with mild decreases in the expression of Bdnf in WT and *Jnk3*^*−/−*^ HFD groups. Meanwhile, *Jnk1*^*−/−*^ CT animals showed mild increases in these same genes, as well as in *Ppp1r9b*, gene that codes for neurabin-2. Also, loss of function of JNK1 favored increases in the different isoforms of neurexin (*Nrxn*) and neuroligin 3 (*Nlgn3*). *Nrxn3* was downregulated by HFD in WT and *Jnk3*^*−/−*^ conditions (Fig. 4A, B; *Bdnf*: *p* < 0.05 WT HFD vs *Jnk1*^*−/−*^ CT and *Jnk1*^*−/−*^ CT vs *Jnk3*^*−/−*^ HFD; *Ntrk2*: *p* < 0.05 WT CT vs WT HFD, WT vs *Jnk1*^*−/−*^ HFD, *Jnk1*^*−/−*^ CT vs *Jnk3*^*−/−*^ HFD, *p* < 0.01 WT HFD vs *Jnk1*^*−/−*^ CT, WT HFD vs *Jnk3*^*−/−*^ CT and *Jnk3*^*−/−*^ CT vs *Jnk3*^*−/−*^ HFD; *p* < 0.001 *Jnk1*^*−/−*^ CT vs *Jnk1*^*−/−*^ HFD and *Jnk1*^*−/−*^ HFD vs *Jnk3*^*−/−*^ CT; *Ppp1r9b*: *p* < 0.05 WT HFD vs *Jnk1*^*−/−*^ CT, *Jnk1*^*−/−*^ CT vs *Jnk1*^*−/−*^ HFD, *Jnk1*^*−/−*^ CT vs *Jnk3*^*−/−*^ CT and *Jnk1*^*−/−*^ CT vs *Jnk3*^*−/−*^ HFD; *p* < 0.01 WT CT vs *Jnk1*^*−/−*^ CT; *Nrxn1*: *p* < 0.05 WT CT vs *Jnk1*^*−/−*^ CT and WT HFD vs *Jnk1*^*−/−*^ CT; *p* < 0.01 *Jnk1*^*−/−*^ CT vs *Jnk1*^*−/−*^ HFD, *Jnk1*^*−/−*^ CT vs *Jnk3*^*−/−*^ CT and *Jnk1*^*−/−*^ CT vs *Jnk3*^*−/−*^ HFD; *Nrxn2*: *p* < 0.05 WT HFD vs *Jnk1*^*−/−*^ CT and *Jnk1*^*−/−*^ CT vs *Jnk3*^*−/−*^ CT; *p* < 0.01 *Jnk1*^*−/−*^ CT vs *Jnk1*^*−/−*^ HFD and *Jnk1*^*−/−*^ CT vs *Jnk3*^*−/−*^ HFD; *Nrxn3*: *p* < 0.05 WT CT vs WT HFD, WT CT vs *Jnk3*^*−/−*^ CT, WT HFD vs *Jnk3*^*−/−*^ CT and WT HFD vs *Jnk3*^*−/−*^ HFD; *p* < 0.01 WT HFD vs *Jnk1*^*−/−*^ CT; *p* < 0.001 WT HFD vs *Jnk1*^*−/−*^ HFD and WT HFD vs *Jnk3*^*−/−*^ CT; *Nlgn3*: *p* < 0.05 WT HFD vs *Jnk1*^*−/−*^ CT and *Jnk1*^*−/−*^ CT vs *Jnk3*^*−/−*^ HFD).

### The pattern of dendritic spines only is maintained in *Jnk1*^−/−^ mice

**Fig. 5:**
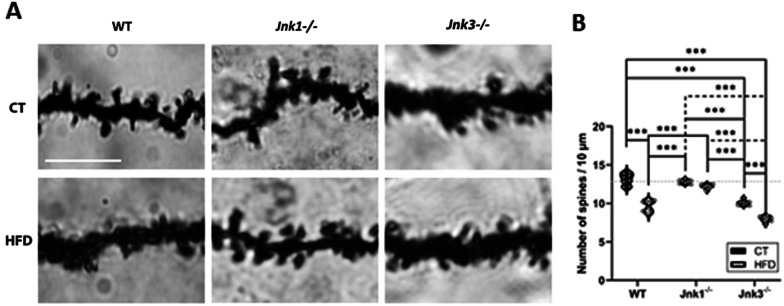
**A** Representative image of the state of the dendritic spines as observed in the optical microscope for each experimental group (Golgi stain). Scale bar: 10 µm. **B** Comparative quantification of the number of dendritic spines in 10 µm sections of the dendritic arborization of neurons of the hippocampal dentate gyrus (n = 8/experimental group). Results were represented as violin plots (**B**). Differences between groups were analyzed using ANOVA and Tukey’s. Significance was represented as follows: *p < 0.05, **p < 0.01 and ***p < 0.001

Quantification of dendritic spines in the ramifications of granular neurons in the dentate gyrus of the hippocampus indicated that WT HFD animals, as well as animals that had a loss of function of JNK3, presented a reduction in the shape, length and number (Fig. 5A, B; *p* < 0.001 WT CT vs WT HFD, WT CT vs *Jnk3*^*−/−*^ CT, WT CT vs *Jnk3*^*−/−*^ HFD, WT HFD vs *Jnk1*^*−/−*^ CT, WT HFD vs *Jnk1*^*−/−*^ HFD, *Jnk1*^*−/−*^ CT vs *Jnk3*^*−/−*^ CT, *Jnk1*^*−/−*^ CT vs *Jnk3*^*−/−*^ HFD, *Jnk1*^*−/−*^ HFD vs *Jnk3*^*−/−*^ CT, *Jnk1*^*−/−*^ HFD vs *Jnk3*^*−/−*^ HFD and *Jnk3*^*−/−*^ CT vs *Jnk3*^*−/−*^ HFD). Animals in the *Jnk1*^*−/−*^ experimental groups showed control-like responses in both analyses (Fig. 5A, B).

### *Jnk3*^−/−^ mice show metabolic stress independently of dietary factors

**Fig. 6:**
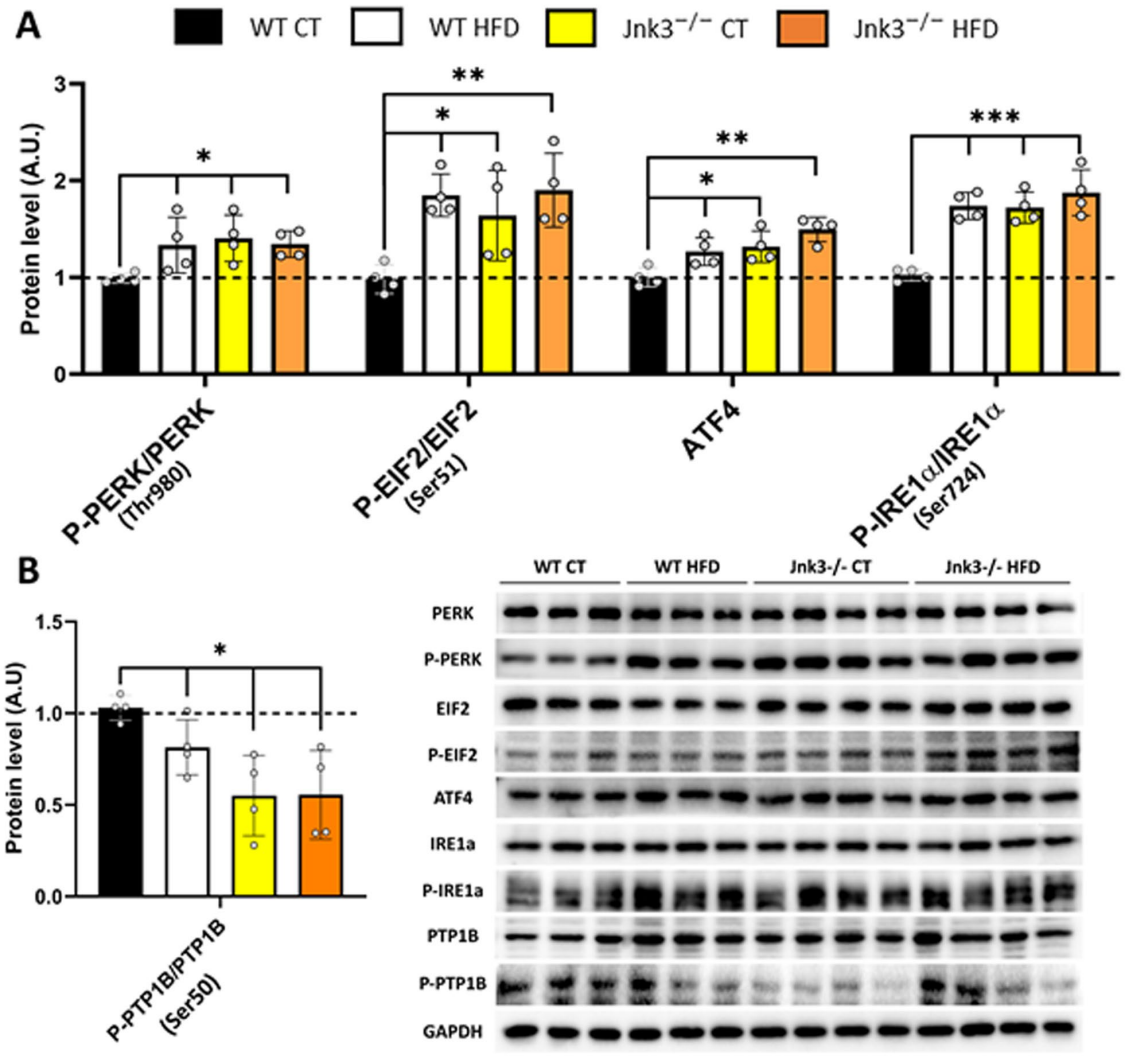
Loss of function of JNK3 promotes dysregulation of normal metabolic function. Protein levels were detected. **A** Endoplasmic reticulum-related [PERK, P-PERK (Thr980), EIF2, P-EIF2 (Ser51), ATF4, IRE1α and P-IRE1α (Ser724)] and **B** PTP1B and P-PTP1B (Ser50). Results were represented as bar graphs and differences between groups was analyzed using ANOVA and Tukey’s (n = 4 per experimental group). Significance was represented as follows: *p < 0.05, **p < 0.01 and ***p < 0.001

Immunoblot detections showed increased levels of total and phosphorylated endoplasmic reticulum stress-related proteins when compared to control conditions: P-PERK/PERK (Thr980) (*p* < 0.05 WT vs WT HFD, *Jnk3*^*−/−*^ CT and *Jnk3*^*−/−*^ HFD), P-EIF2/EIF2 (Ser51) (*p* < 0.05 WT vs WT HFD, *Jnk3*^*−/−*^ CT and *p* < 0.01 WT vs *Jnk3*^*−/−*^ HFD), ATF4 (*p* < 0.05 WT vs WT HFD, *Jnk3*^*−/−*^ CT and *p* < 0.01 WT vs *Jnk3*^*−/−*^ HFD) and P-IRE1α/ IRE1α (Ser724) (*p* < 0.001 WT vs WT HFD, *Jnk3*^*−/−*^ CT and *Jnk3*^*−/−*^ HFD) (Fig. 6A). Detection of the protein PTP1B indicated a decrease in the levels of the total and phosphorylated forms (Ser50) (*p* < 0.05 WT vs WT HFD, *Jnk3*^*−/−*^ CT and *Jnk3*^*−/−*^ HFD) (Fig. 6B).

### *Jnk3*^−/−^ and *Jnk1*^−/−^ mice are protected against astrocytosis

**Fig. 7:**
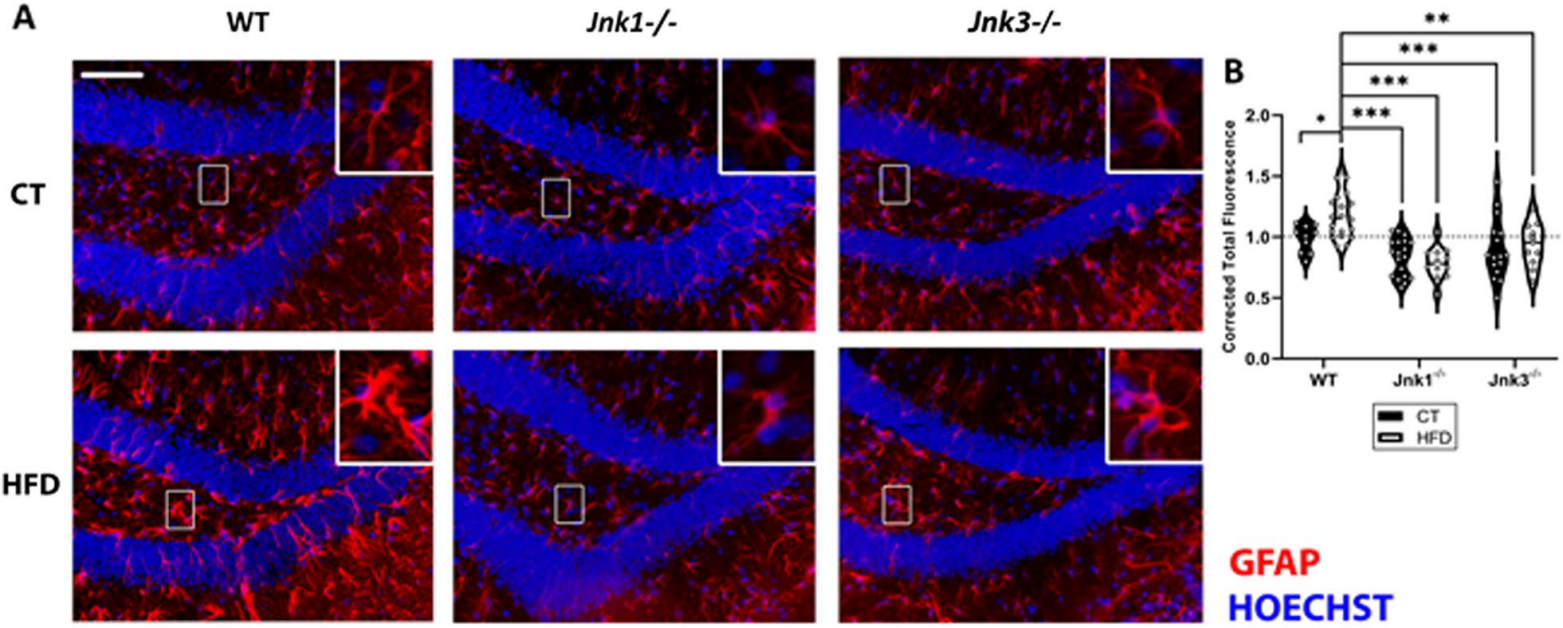
**A** Representative images of GFAP-labeling (red) in the dentate gyrus of the hippocampus. Hoechst was used to stain the nuclei (blue). Scale bar: 100 µm (n ≥ 15). **B** Calculation of differences in fluorescence intensity for GFAP. Relative intensity quantification numbers were obtained under the following formula: CTCF (Corrected Total Cell Fluorescence) = Integrated Density − (Area of selected cell X Mean fluorescence of background readings). Differences between groups were analyzed using ANOVA and Tukey’s. Significance was represented as follows: *p < 0.05, **p < 0.01 and ***p < 0.001 (n = 4/experimental group)

HFD increased the reactivity of astrocytes in WT conditions, but not in *Jnk1*^*−/−*^ and *Jnk3*^*−/−*^ mice (Fig. 7A, B; *p* < 0.05 WT CT vs WT HFD; *p* < 0.01 WT HFD vs *Jnk3*^*−/−*^ HFD; *p* < 0.001 WT HFD vs *Jnk1*^*−/−*^ CT, WT HFD vs *Jnk1*^*−/−*^ HFD and WT HFD vs *Jnk3*^*−/−*^ CT).

## Discussion

Published data on the loss of function on JNK3 has described its beneficial effects in scenarios of cellular death and cytotoxicity, just like its importance in brain development in the embryonic stages (Yang et al. 1997; Brecht et al. 2005) but, not much data has been gathered on the consequences of its ablation in bioenergetics of the brain. The only published evidence is that of Vernia et al., who demonstrated that metabolic stress induces hyperphagia and severe obesity in JNK3 KO mice due to its essential role in the control of the response to leptin in the hypothalamus (Vernia et al. 2016). In our attempt to understand better the role of JNK3, we analyzed the consequences of the loss of function of JNK3 in the endoplasmic reticulum. In our observations we detected significantly increased protein levels of elements linked to organelle stress independently of the HFD (Busquets et al. 2019a; Raciti et al. 2012). Additionally, we checked for changes in levels of PTP1B, a regulatory element of the activity of insulin, leptin and BDNF receptors. Results indicated an increased activation state of this phosphatase in all experimental conditions. Increases in the activity of PTP1B, just like stress in the endoplasmic reticulum, have been associated with metabolic disturbances both in the periphery and central areas and, have been linked with a diverse array of pathological states, including neurodegenerative diseases (Thiebaut et al. 2016; Ono 2019; Chen et al. 2015; Verma and Sharma 2018; Ravichandran et al. 2001). When put together, these two results would be evidence to the dysregulated metabolic state of JNK3 KO animals as indicated by Vernia et al.

The next part of the study focused on variations of the expression of an array of genes linked to metabolic function and cognitive performance. This was carried out by making a comparison of controls against samples of animals that had been exposed to chronic metabolic stress (HFD) and, combined by a loss of function of JNK3 or JNK1 which, in a previous study had shown to be highly efficient metabolically and to be resistance to the consequences of HFD (Busquets et al. 2019b). Data from the array allowed us to raise several points: (1) Chronic HFD feeding caused disruption in energetic metabolic pathways. This would be in accordance with previous reports in the field in which HFD has been demonstrated to disrupt physiological metabolism and promote insulin resistance, as well as obesity and other pathological alterations (Tsan et al. 2021; Leigh and Morris 2020; Penna et al. 2020). Increased ingestion of fats, obesity and resistance to hormones like insulin caused disturbances in normal cognitive function and structures as reported by our research team and others (Tsan et al. 2021; Leigh and Morris 2020; Penna et al. 2020). (2) Combination of HFD and KO of JNK3 led to synergetic responses. Animals showed increased body weight, especially when exposed to a HFD as described by Vernia et al. (2016); also, they had significantly decreased response to insulin. Additionally, increased stress in the endoplasmic reticulum and diminished cognitive capacity were detected. (3) Loss of function of JNK1 promoted normal or heightened energetic metabolism, paired with an increase in the expression of antioxidant enzymes; which would allow for a reduction in the load of oxidative species; an essential mechanism in the brain (Cobley et al. 2018). Animals that lacked JNK1 weighed less and were more sensitive to insulin even when fed chronically with HFD. This data correlated with previous reports from our own research and that of other laboratories (Sabio et al. 2009, 2010; Busquets et al. 2019b; Jodeiri Farshbaf et al. 2016; Grivennikov et al. 2007; Belgardt et al. 2010). Furthermore, the loss of function of JNK1 preserved cognitive function even when chronically metabolically stressed by showing higher BDNF, BDNF receptor and neurabin expression. Maintenance of proper synaptic connections in the brain though neuronal physical connections between adhesion molecules which promote pre- and post-synaptic organization for neurotransmitter release and reception is indispensable for normal cognitive function (Ribeiro et al. 2019). In the postsynaptic region, neuroligins interact with neurexins to maintain synaptic communication. In our study, the hippocampal mRNA levels of all three neurexin isoforms were higher in *Jnk1*^*−/−*^ CT mice.

Whereas loss of function of JNK1 promotes normal or heightened energetic metabolism, paired with an increase in the expression of antioxidant enzymes *Sod1* and *Gpx1*, knockout of JNK3 causes the downregulation of glucose transporter *Slc2a1*. It is well known that the brain and, specifically, neurons have a high energy requirement, therefore they depend on the availability and use of glucose (Cobley et al. 2018). Our results indicate a significant decrease in mRNA levels in GLUT1 expression in the hippocampus of *Jnk3*^*−/−*^ mice compared to *jnk1*^*−/−*^ mice. The effects of the HFD exacerbated this decrease. Likewise, the hippocampal expression of GLUT1 is associated with the process of learning and memory (Jodeiri Farshbaf et al. 2016). Therefore, lower mRNA GLUT1 levels could lead to insufficient energy supply and perturbation of neuronal function in the *Jnk3*^*−/−*^ mice brain. In addition, our data demonstrated a decrease in mRNA levels complex I, III, V in jnk*3*^*−/−*^ mice associated with HFD. Likewise, we found a significant decrease in mRNA levels of Uqcrc1, which is involved in the formation of mitochondrial complex III. Although the exact function of *Uqcrc1* is unknown, it was reported that in the heterozygous *Uqcrc1*^+/−^ mice this mitochondrial protein plays a critical role in maintaining brain functions among them a key cognitive role and its loss favors a decrease in ATP production and ROS increase.

In this process of oxidative phosphorylation at the mitochondrial level, ROS are also generated as a by-product. However, as we have previously discussed, *Jnk1*^*−/−*^ mice have higher levels of mRNA from antioxidant enzymes such as SOD and GSH which can protect from the potential adverse effects of ROS. In addition, these mice have higher expression of the GLUT1 mRNA, with which these mice could have a better brain energy intake that would be accompanied by a better functionality of the insulin receptor-signaling pathway at the level of hippocampus. Therefore, in all this process where ATP is synthesized, we can conclude that the hippocampal energy state of the *Jnk1*^*−/−*^ mice will be much better than that of *Jnk3*^*−/−*^ and this process is related to the modulation of neurotransmitters such as glutamate and GABA, which have a key role in the process of synaptic plasticity (Belgardt et al. 2010). All this data would be evidence to the idea that maintenance of a healthy energetic metabolism may prevent and/or delay the development of the initial stages of mild cognitive impairments (Chen et al. 2015; Verma and Sharma 2018; Ravichandran et al. 2001; Wang et al. 2020; Tsan et al. 2021; Leigh and Morris 2020; Penna et al. 2020; Cobley et al. 2018). The beneficial effects of a partial loss of function of JNK1 in a genetic model of Alzheimer’s disease (APP/PS1/*Jnk1*^±^) was reported in the past by our research group (Petrov et al. 2015).

Observation and quantification of the reactive profile of astrocytes reproduced the increased reactivity of these cells in WT animals when fed chronically with HFD (Busquets et al. 2017; Ettcheto et al. 2017), another mechanism through which neurodegeneration occurs (Chitnis and Weiner 2017). The loss of function of either of the JNK isoforms prevented this effect due to their direct involvement in the mounting of inflammatory responses (Sabapathy 2012; Cui et al. 2007).

## Conclusions

Loss of function of JNK3 does not seem to be a suitable target for the development of therapeutic approaches due to its detrimental effects in the control of metabolism and cognitive function, despite its described effects in the prevention of activation of inflammatory and apoptotic pathways. Yet, the downregulation of JNK1 for the modulation of energetic metabolism proves to be a very attractive approach for the amelioration of cognitive-neurodegenerative states linked to insulin resistance and increased tissue inflammation. This study may allow a better understanding of the role of both isoforms in the brain and their potential role in the treatment of neurological diseases (Additional files 2, 3, 4, 5, and 6).

## Supplementary Information

**Additional file 1: Additional Material 1.** Specific descriptions of the genes included in the TaqMan® array.

**Additional file 2.** **Sup. Fig 2.** Hematoxylin-eosin stain in different hippocampal areas.

**Additional file 3.** **Sup. Fig 3.** Annex is show that no significant differences were observed in the levels of GADPH and tubulin in any of the experimental conditions.

**Additional file 4.** **Sup Fig 4.**Determination of the levels of Superoxide dismutase, Catalase and 4-Hydroxynonenal in the hippocampus of JNK3-/- mice treated with a control diet and a high-fat diet

**Additional file 5.** **Sup Fig 5.** Expression of JNK1 and JNK3 levels in the hippocampus of mice treated with a standard diet and treated with a high-fat diet.

**Additional file 6. **Immunoblot membranes.

## Data Availability

The data sets used and/or analyzed during the current study are available from the corresponding author on reasonable request.

## Abbreviations

JNK: C-Jun N-terminal Kinases
MAPK: Mitogen Activated Protein Kinases
IRE1α: Inositol-Requiring Enzyme 1 alpha
PERK: Protein Kinase-like Endoplasmic Reticulum Kinase
EIF2: Eukaryotic Initiation Factor 2 α
ATF4: Activated Transcription Factor 4
PTP1B: Protein Tyrosine Phosphatase 1 Beta
HFD: High-fat diet
NORT: Novel object recognition test
ITT: Insulin tolerance test
PFA: Paraformaldehyde
FBS: Fetal bovine serum
GFAP: Glial fibrillary acidic protein
DG: Dentate gyrus
Nrxn: Neurexin
Nlgn: Neuroligin
