# Supplementary material for: JNK1 and JNK3: divergent functions in hippocampal metabolic-cognitive function
Source: Mol Med. 2022 May 4;28:48. doi: 10.1186/s10020-022-00471-y (PMC9066854; doi:10.1186/s10020-022-00471-y)
Supplement: Supplementary file 1 — Additional file 1: Additional Material 1. Specific descriptions of the genes included in the TaqMan® array. [file 10020_2022_471_MOESM1_ESM.pdf]

Additional Material 1. **Specific descriptions of the genes included in the TaqMan® array.**

**Gene**	**Full Protein Name**
Slc2a1	Glucose Transporter Type 1 (GLUT1)
Slc2a2	Glucose Transporter Type 2 (GLUT2)
Slc2a3	Glucose Transporter Type 3 (GLUT3)
Slc2a4	Glucose Transporter Type 4 (GLUT4)
Insr	Insulin Receptor (IR)
Irs1	Insulin Receptor Substrate 1 (IRS1)
Irs2	Insulin Receptor Substrate 2 (IRS2)
Prkaa1	Protein Kinase AMP-Activated Catalytic Subunit Alpha 1 (AMPK)
Akt1	Protein Kinase B (AKT)
Akt2	Protein Kinase B (AKT)
Creb1	CAMP Responsive Element Binding Protein 1 (CREB1)
Gsk3β	Glycogen Synthase Kinase 3 Beta (GSK3β)
Pparγ	Peroxisome proliferator-activated receptor gamma (PPARγ)
Pparγc1α	Peroxisome proliferator-activated receptor gamma coactivator 1-alpha (PGC1α)
Ptpn1	Protein tyrosine phosphatase 1B (PTP1B)
Hk1	Hexokinase 1 (HK1)
Hk2	Hexokinase 2 (HK2)
Pfkp	Phosphofructokinase (PFK)
Pkm	Pyruvate kinase (PK)
Pdha1	Pyruvate dehydrogenase subunit 1 (PDHA1)
Pdha2	Pyruvate dehydrogenase subunit 2 (PDHA2)
Ndufv1	NADH dehydrogenase ubiquinone flavoprotein 1 (NDUFV1; subunit of OXPHOS CI)
Sdha	Succinate Dehydrogenase Complex Flavoprotein Subunit A (SDHA; subunit of OXPHOS CII)
Sdhb	Succinate Dehydrogenase Complex Flavoprotein Subunit B (SDHB; subunit of OXPHOS CII)
Uqcrc1	Ubiquinol-Cytochrome C Reductase Core Protein 1 (UQCRC1; subunit of OXPHOS CIII)
Uqcrb	Ubiquinol-cytochrome c reductase binding protein (UQCRB; subunit of OXPHOS CIII)
Cycs	Cytochrome C (CYC)
Cox4i1	Cytochrome C Oxidase Subunit 4I1 (COX4i1; subunit of OXPHOS CIV)
Atp5b	ATP synthase F1 subunit beta (ATP5B; subunit of OXPHOS CV)
Sod1	Superoxide dismutase 1 (SOD1)
Gpx1	Glutathione peroxidase 1 (GPX1)
Cat	Catalase (CAT)
Bdnf	Brain-Derived Neurotrophic Factor (BDNF)
Ntrk2	Neurotrophic Receptor Tyrosine Kinase 2 (BDNFR)
Ppp1r9b	Protein Phosphatase 1 Regulatory Subunit 9B – Neurabin2 (NRBN)
Syp	Synaptophysin (SYP)
Dlg4	Postsynaptic density protein 95 (PSD95)
Nrxn1	Neurexin 1 (NRXN1)
Nrxn2	Neurexin 2 (NRXN2)
Nrxn3	Neurexin 3 (NRXN3)
Nlgn1	Neuroligin 1 (NLGN1)
Nlgn2	Neuroligin 2 (NLGN2)
Nlgn3	Neuroligin 3 (NLGN3)
