# Supplementary figures and images for: JNK1 and JNK3: divergent functions in hippocampal metabolic-cognitive function
Source: Mol Med. 2022 May 4;28:48. doi: 10.1186/s10020-022-00471-y (PMC9066854; doi:10.1186/s10020-022-00471-y)

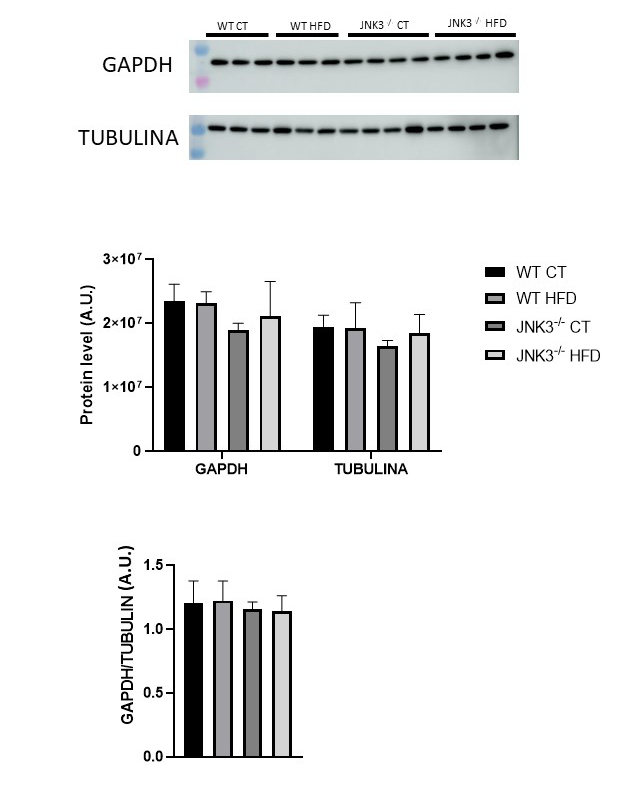

Supplement: Supplementary file 3 — Additional file 3. Sup. Fig 3. Annex is show that no significant differences were observed in the levels of GADPH and tubulin in any of the experimental conditions. [file 10020_2022_471_MOESM3_ESM.tif]

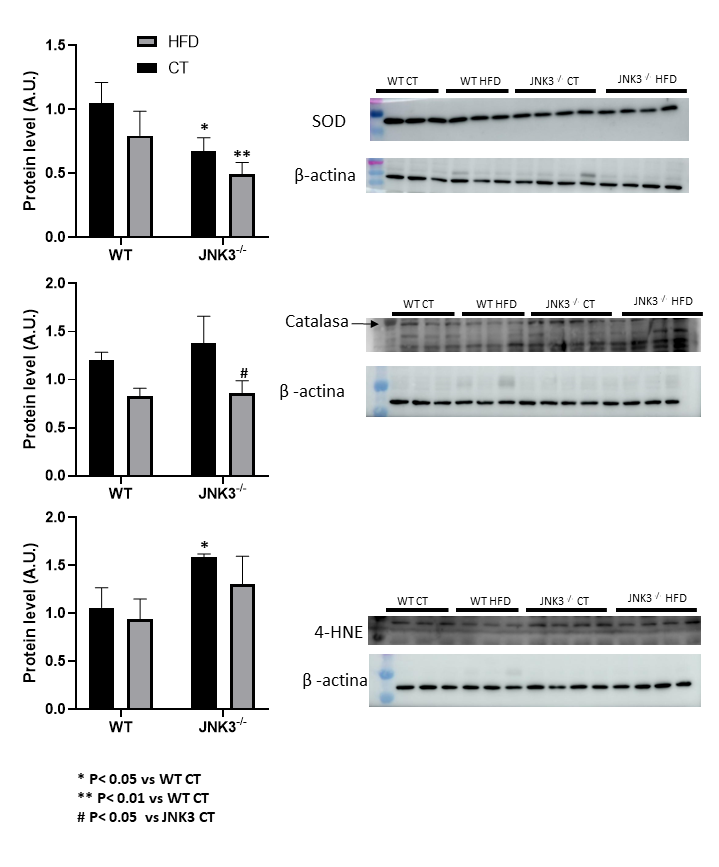

Supplement: Supplementary file 4 — Additional file 4. Sup Fig 4.Determination of the levels of Superoxide dismutase, Catalase and 4-Hydroxynonenal in the hippocampus of JNK3-/- mice treated with a control diet and a high-fat diet [file 10020_2022_471_MOESM4_ESM.tif]

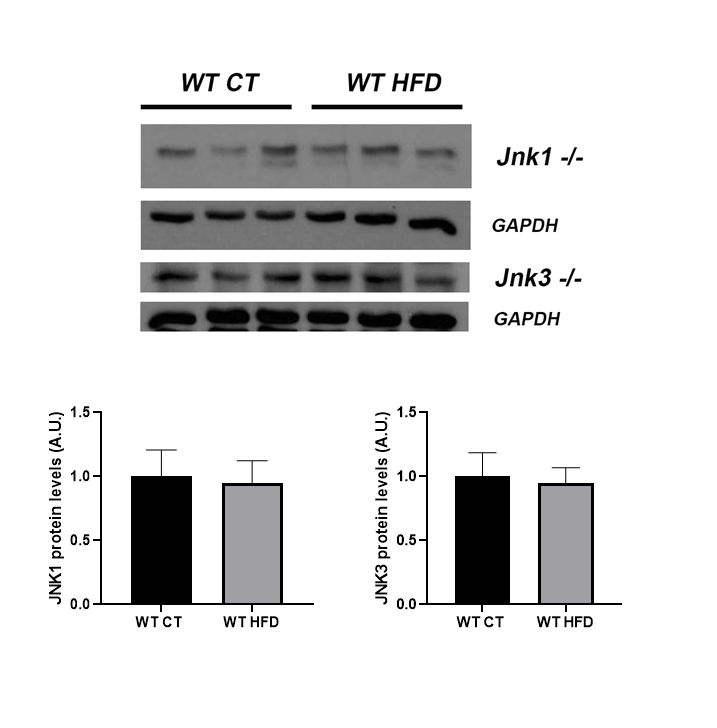

Supplement: Supplementary file 5 — Additional file 5. Sup Fig 5. Expression of JNK1 and JNK3 levels in the hippocampus of mice treated with a standard diet and treated with a high-fat diet. [file 10020_2022_471_MOESM5_ESM.tif]
