# Supplementary material for: JNK1 and JNK3: divergent functions in hippocampal metabolic-cognitive function
Source: Mol Med. 2022 May 4;28:48. doi: 10.1186/s10020-022-00471-y (PMC9066854; doi:10.1186/s10020-022-00471-y)
Supplement: Supplementary file 6 — Additional file 6. Immunoblot membranes. [file 10020_2022_471_MOESM6_ESM.pdf]

Additiona1 materia1: Immunoblot Membranes.

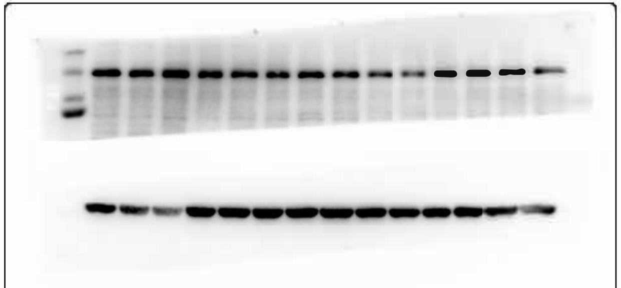

PERK + TUBULIN

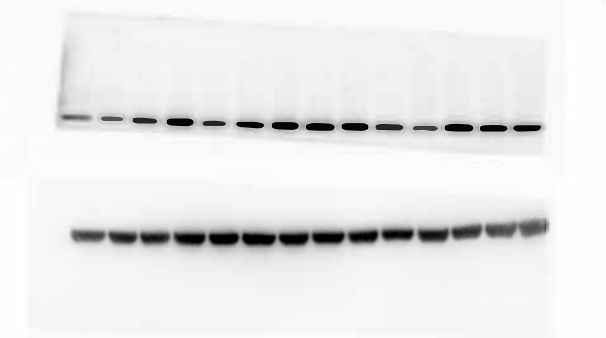

P-PERK + TUBULIN

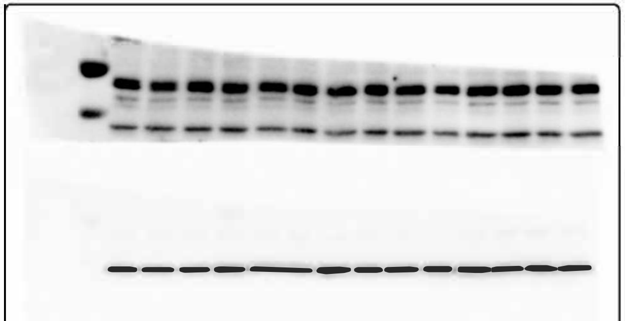

ATF4 + GAPDH

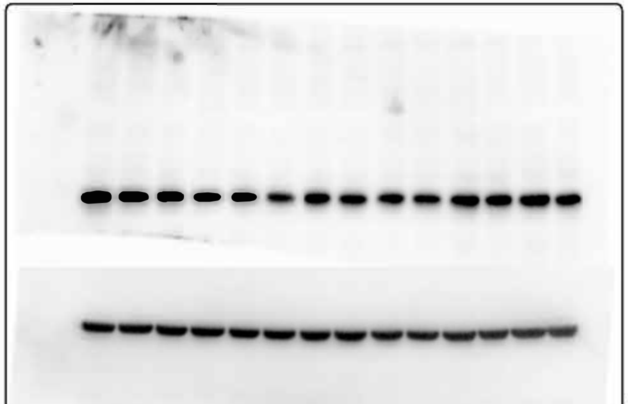

EIF2 + TUBULIN

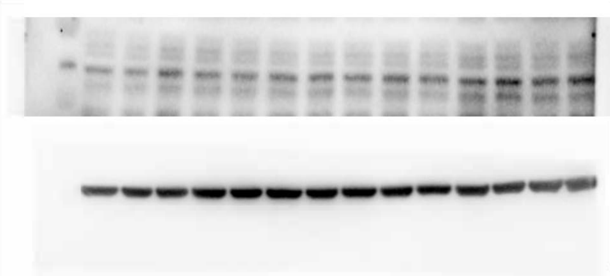

P-EIF2 + TUBULIN

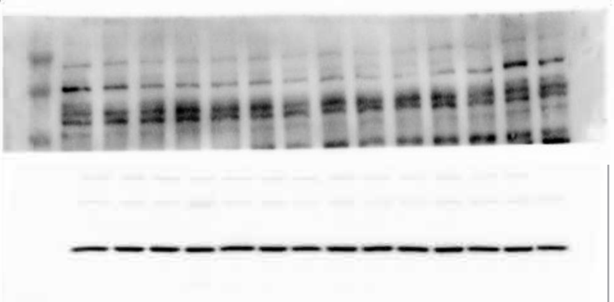

P-IRE1a + GAPDH

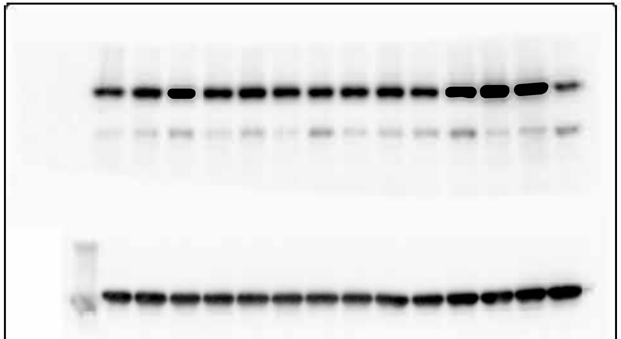

IRE1a + GAPDH

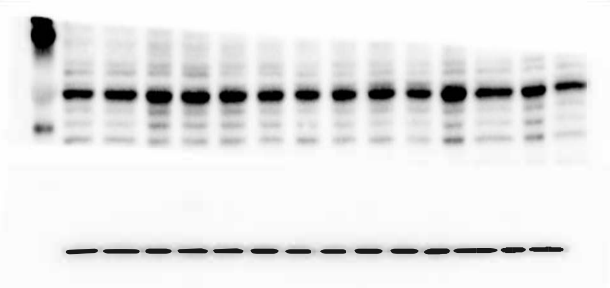

PTP1B + GAPDH

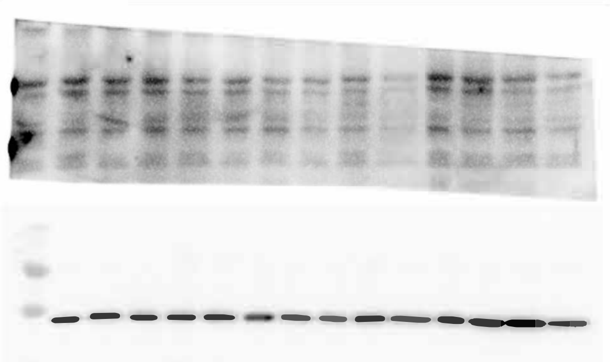

P-PTP1B + GAPDH
